# Real-Time Reliability Verification for UAV Flight Control System Supporting Airworthiness Certification

**DOI:** 10.1371/journal.pone.0167168

**Published:** 2016-12-05

**Authors:** Haiyang Xu, Ping Wang

**Affiliations:** 1 School of Science and Information, Qingdao Agricultural University, Qingdao, Shandong, China; 2 School of Computer Science and Technology, Nanjing University of Aeronautics and Astronautics, Nanjing, Jiangsu, China; West Virginia University, UNITED STATES

## Abstract

In order to verify the real-time reliability of unmanned aerial vehicle (UAV) flight control system and comply with the airworthiness certification standard, we proposed a model-based integration framework for modeling and verification of time property. Combining with the advantages of MARTE, this framework uses class diagram to create the static model of software system, and utilizes state chart to create the dynamic model. In term of the defined transformation rules, the MARTE model could be transformed to formal integrated model, and the different part of the model could also be verified by using existing formal tools. For the real-time specifications of software system, we also proposed a generating algorithm for temporal logic formula, which could automatically extract real-time property from time-sensitive live sequence chart (TLSC). Finally, we modeled the simplified flight control system of UAV to check its real-time property. The results showed that the framework could be used to create the system model, as well as precisely analyze and verify the real-time reliability of UAV flight control system.

## 1. Introduction

UAV has been used in a vast range of civil and military applications, and also brought accidents caused by airborne software failure[1–3], we expect to develop a high reliable UAV flight control system. The traditional approach used for manned aircrafts takes robust time and resources, which is not practical to analyze and validate UAV flight control system. For shortening the development cycle and improve the reliability and performance of flight control system, developing an integrated framework for the design process of flight control system is in need[4]. This framework could integrate the existing design methods and verification tools, and use iterative development cycle to implement and quickly validate UAV flight control system design. Therefore, it is important to enhance the reliability and robustness of UAV flight control system by improving the method of modeling, testing and verifying.

Federal Aviation Administration (FAA) requires that the airborne software system must be conducted by airworthiness certification[5]. Thus, we should model and verify the UAV flight control system in term of corresponding airworthiness certification standards.

In airworthiness certification, we considered the DO-178B standard, which is the current software certification standard that released by Radio Technical Commission for Aeronautics (RTCA). DO-178B prescribes the objectives for each important step during the development process of airborne software[6]. With the development of the software verification technology, RTCA and European Organization for Civil Aviation Equipment (EUROCAE) revised the DO-178B and published the DO-178C/ED-12C standard in 2011. DO-178C/ED-12C proposed several technical supplements such as software tool qualification considerations, model-based development and verification, object-oriented technology and formalizing methods [7–9].

Development and verification based on executable models can optimize the process of airborne software. During the stage of requirement and design, we should find out the software fault as early as possible in order to eliminate the errors of design and enhance the robustness of software. According to DO-178C/ED-12C, source code should be generated from design model directly, and they all should pass the validation.

In the development of airborne software, object-oriented technology benefits the generation of source code for test and certification via model driven architecture (MDA) or MARTE tool. It can improve the reusability and validity of software. DO-178C/ED-12C therefore requires adopting object-oriented technology.

DO-l78C officially indicated the effectiveness of formal methods during airborne software development process. Formal method is consisted of formal model and formal analysis. Formal model is used for defining unambiguous abstract model of system based on mathematic syntax and semantic. Formal analysis proved the consistency between system property and software requirement by theorem proving or model checking.

The design and verification of airborne software should comply with the guidance of DO-178C to obtain certification approval[10]. Recently, in order to improve the development method of airborne software and meet airworthiness certification, researchers have proposed several integrated development frameworks. Most of these methods focus on the model-based development environment to make different tools and techniques adopted an applied. The main challenge of model-based development approach is that we should generate precision appropriate dynamic models of airborne software at different development stages[1]. Mathematical model can only be used to describe simplified real flight control, and is just an approximation of the airborne software, because sensors of UAV are inaccurate and aerodynamic data are not understandable enough. Therefore, mathematical model is not be able to be used for predicting the real-time reliability responses.

To study the feasibility of applying model checking to avionic embedded software, Sreemani et al. took advantage of software cost reduction to describe software requirement specification of military aircraft A-7E, and then translated specification to symbolic model verifier (SMV)[11]. They pointed out that model checking was of help to provide a safe and reliable software, but it could not be extended to large-scale software.

To verify the requirement specification of large-scale software, Chan et al. proposed an interactive design process based on the progressive refinement of the models. This process can specify the set of properties, and adopt SMV to check the requirements specification of TCAS II. The limitation is that model checkers cannot be integrated with other CASE tools.

Pingree et al. applied model checking to the development of flight software for NASA’s Deep Space 1 mission[12]. They used State flow to create software model and translated it into SPIN. The approach can automatically generate software code from State chart model, but they cannot assure that all design errors could be showed in the model.

For rapidly synthesize, analyze and validate a candidate UAV software design, Yew Chai Paw et al. proposed a model-based framework, which integrates a set of design tools to realize software model synthesis, off-line and real-time simulation[1]. They pointed out that simulation based on tests is important for saving cost and time. However, Seveg et al. compared simulation with formal verification in SoCs design process[13]. They indicated that formal verification requires more time and memory, but it can identify failed properties and get the highest credibility of the verified system.

Cofer et al. inserted formal analysis tools into a model-based development process to improve quality of avionics software[14]. They used State flow to generate model-based specification, and used Lustre as an intermediate representation for the models, and then translated specification into NuSMV model checker. They applied the method to the FCS 5000 flight control system and an adaptive flight control system for UAV[15].By analyzing the causes of 156 failures on 129 space crafts, Tafazoli pointed out that 6% of these failures are due to software failure[16].

In this study, our purpose is to verify real-time reliability for UAV flight control system. Combining model-based method with formal analysis tool, we can reduce the cost of building separate analysis models, and keep the model consistent with the software design.

Combining with the requirements of DO-l78C, we utilized MARTE class diagrams to set up a time-related static model of flight control system based on MDA. We also employed dynamic models triggered by time to describe system state changes. As MARTE is a graphical model, we proposed a formal PTA-OZ model and constructed its model transformation rules. Using these rules, we can translate both static models and dynamic models into corresponding Object-Z model and PTA models, which are fit for system real-time reliability verification and code automatic generation. In order to get the real-time specification of design model, we presented a method to extract real-time property from TLSC.

This paper proceeds as follows. Section 2 outlines the logic structure of flight control system. Section 3 describes the PTA-OZ model based on MDA. Section 4 gives the real-time property extract method from scenario-based language. Section 5 reports a case study of real-time reliability verification. Finally, Section 6 concludes the paper.

## 2. The Logic Structure of Flight Control System

The software architecture of UAV flight control system mainly consists of communication link (CL) module, sensor signal processing module, flight guidance (FG) module, servo control (SC) module, mode supervise (MS) module and PWM steering output modules.

1. CL module is used for receiving telecommand (TC) from the ground control station (GCS), down-transporting telemetry (TM) to GCS and for data communication among the airborne modules.
2. The function of FG module includes control law computing, TC verification and response, route planning, operator scheduling and sending control command.
3. SC module is applied to response control command and send servo control.
4. MS module is mainly used for conflict detection and conflict resolution.

**Fig 1:**
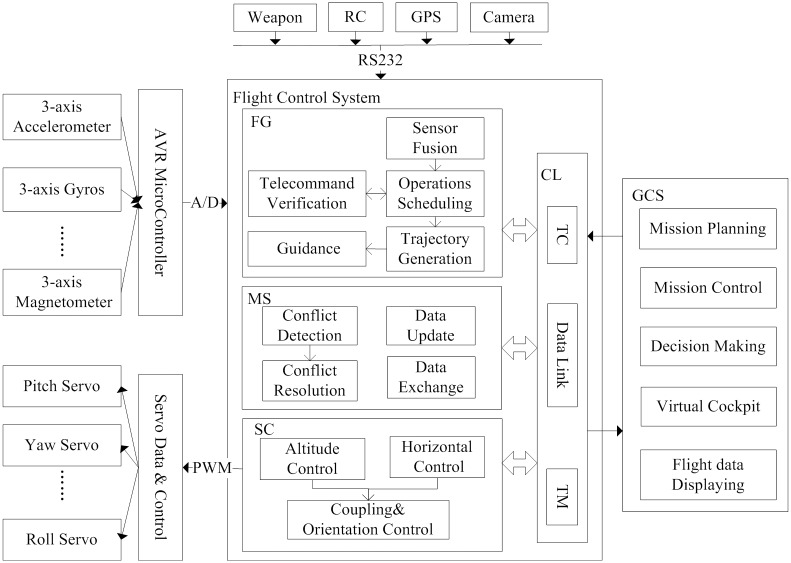
Part of hardware and software architecture of the flight control system. The figure shows parts of the organizational structure of a flight control system, and describes the data interaction among module.

The flight control system uses embedded system design which is based on PC104 bus. It includes five main components: processor module, serial port module, A/D, D/A card module and power module. The communication among modules uses PC104 bus. Processor module communicates with FG module and SC module by adopting TCP/UDP protocol. Serial port module uses RS232 protocol to communicate with sensor module in serial communication. The system uses A/D of AVR to collect signals, such as six degree of freedom information and supply voltage, and uses D/A card to output PWM signal. Fig 1 shows part of hardware and software architecture of the flight control system.

We focused on the commands and data processing structure among GCS and FG module, SC module, and MS module. We not only require the UAV flight control software to satisfy the DO-178C airworthiness certification requirements, but also fully reference ECSS-E-70-41A standard to standardize the service offered by each module. So we employed telecomm and verification service and onboard operations scheduling service which adopt ECSS-E-70-41A standard in FG module of flight control software.

## 3. Modeling and Description of Time Property

The advantages of development method based on MDA in the design process of embedded software are as follows. Firstly, the execution platform of embedded software is usually heterogeneous and reconfigurable. Secondly, applications often need to react with an external environment, and embedded software design focuses on handling data or control flow. Finally, software design is often subject to real-time requirements and resources availability[17]. Above all, in the early stage of embedded software design, the development method based on MDA could detect if resources and services meet the requirements specification under the given constraints.

### 3.1 Time static model based on MARTE

Although model-based development tool such as SCADE suite has been used to formal verify critical avionics software[15], it is difficult to generate code from time synchronism system. SCADE employs a reference or master clock to define all clocks as a functional sample of the master clock[18]. It can provide a solution for generating code in uniprocessor system. However, it is difficult to generate distributed system code. For parallel implementation, MARTE can generate the multi-threaded code from concurrent specification[19].

MARTE, which could be used to establish formal model of real-time and embedded system (RTES), is a new UML extension profile. MARTE defines a mathematically expressive model of time to annotate UML diagrams with formal timing interpretation. MARTE also defines the necessary concepts to build software model of HW/SW embedded system, and its performance depends on the interaction among the different components.

Although UML2 introduces *SimpleTime* package to create time model, it is too simple for RTES. The time models based on MARTE are more suitable for software design. They may be physical, logical, or user-defined. MARTE uses a collection of clocks to represent time, and each clock specifies a totally ordered set of instant.

**Fig 2:**
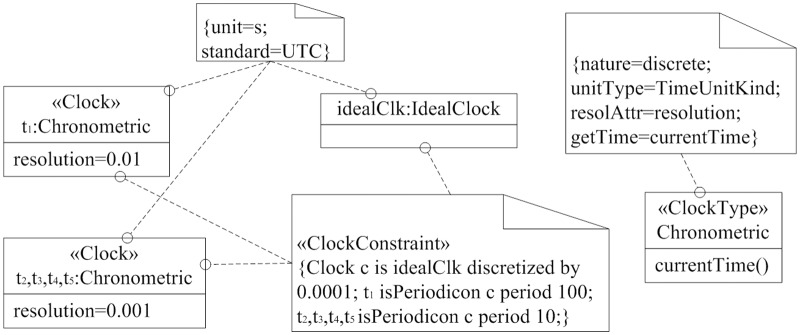
ClockType and its clock. We can use MARTE time stereotypes to define and describe clock class.

In MARTE, «*ClockType*» and «*Clock*» stereotypes can be used to represent the concept of clock. «*ClockType*», which is the type of «*Clock*», specifies common features shared by a family of clocks. «*Clock*» includes more detailed information. We adopted above stereotypes to define a «*ClockType*» and several clock instants (Fig 2).

Firstly, we use «*ClockType*» stereotype to define a new clock type, and specify the feature of clock type with tagged value. The new discrete «*ClockType*» *Chronometric*, whose supportive unite is *s*, uses a readable *resolution* to determine the resolution of the associated clock, and uses *currentTime* operation to get the current time.

Secondly, we introduced a predefine clock *idealClk* in MARTE library, which is an instance of *IdealClock*. It represents the continuous clock of physical time, and uses *s* as its unit of time. *t*_1_, *t*_2_, *t*_3_, *t*_4_, *t*_5_, which are instances of *Chronometric*, use clock constraint to specify their deviations with respect to the ideal one. In «ClockConstraint» stereotype, we adopted clock constraint specification language(CCSL) to express the clock constraints. The *c* is defined as an ideal discrete clock whose resolution is 0.001 *s*. For idealClk, we declare a clock *t*_1_ with a period of 10 *ms* and resolution of 0.01 *s*. The *t*_2_, *t*_3_, *t*_4_, *t*_5_, whose resolution is 0.001 *s*, can be sampled every 10 periods.

**Fig 3:**
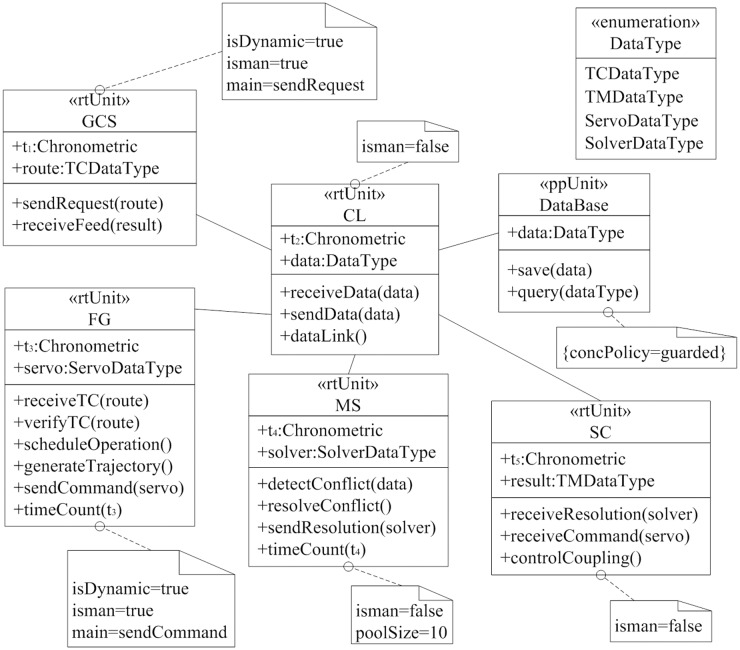
The software logic structure of flight control system. Using RtUnited and PpUnit, we build part of the software logic modle of flight control system.

As MARTE can support system-level design, we adopted RtUnited and PpUnit for the active object of UML to create model of flight control system. Fig 3 showed the class diagrams in software logic structure of flight control system. The structure consists of GCS and airborne system. The logic entities of airborne system include CL module, FG module, SC module and MS module. GCS interacts with airborne system through TC and TM signal. RtUnit is used to represent the entities, which can encapsulate object and behavior in a single entity. Any real-time unit can invoke services of other real-time units to send/receive data flow. In class diagrams, we defined attributes, operations and associations, and offered the interface definitions with entity. FG module can dynamically create schedulable resources to execute its services, and MS module has a pool of 10 schedulable resources.

All real-time units share data that is represented by the class *DataBase*. As the concurrent execution of real-time units, we need to protect the data access encapsulated in the class *DataBase*. In order to do this, we tagged the class *DataBase* by «*PpUnit*» stereotype, whose concPolicy property of *DataBase* is set to guarded, which means that real-time unit should access the *DataBase* property one after another.

### 3.2 Dynamic model supporting time-triggered

State chart can describe the dynamic behaviors of object through creating object model about its life cycle, and focuses on the object behavior changes caused by events. In state chart, an event is an occurrence of motivation, which can trigger state transition. As a special event,Time event represents the state transition triggered by time related factors.

According to the clocks defined in 3.1, we described all kinds of time-triggered mechanisms to create UML dynamic model. So we need to extend UML state chart with time model to support events and behaviors triggered by time.

To reference time-related concept for state chart, we use «*TimedProcessing*» stereotype of MARTE to specify duration for a behavior and improve the use of UML behavior. Through reference defined clocks, we specified behavior of state chart with user-defined clocks to support time-triggered dynamic mechanism, and described RTES software to offer support for multi-clock mechanism of distributed environment.

In MARTE, we use «*TimedEvent*» stereotype to represent time event, which extends from the meta-class *SimpleTime*::*TimeEvent* of UML. Time event is used to specify the state transition triggered by time in state chart.

Flight control system is forced to obey the following operation rules:

1. GCS_request: GCS requests a flight command.
2. FG_command: FG sends a flight command to SC.
3. MS_command: If a flight conflict is detected, MS would send a specific flight command to SC to resolve the flight conflict. MS command is able to override the GCS request and FG command.
4. MS_clear: Executes GCS request or FG command when there is no potential flight conflict. All previous requests would be canceled when a flight conflict was confirmed. At this point, the value of MS_clear variable is false, and the values of all command variables are false.

**Fig 4:**
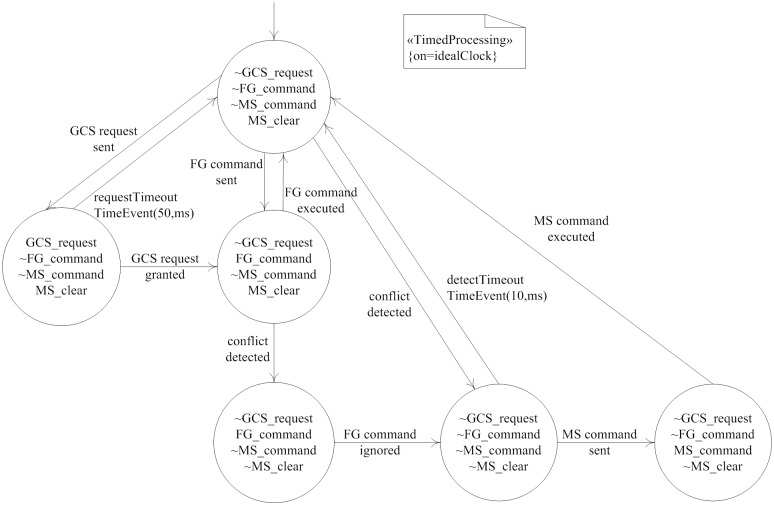
State chart of flight control system. State chart can be used to describe dynamic behavior triggered by event.

Fig 4 described the state chart of flight control system. «*TimedProcessing*» stereotype showed that the dynamic model supports time-triggered mechanism, and use *on* attribute to specify associated clock of the current model. « *TimedEvent*» stereotype was used to define time events: *requestTimeout* and *detectTimeout*. Event *requestTimeout* described that FG module must generate a new air route within 50ms after receiving user’s TC command, otherwise it would trigger timeout transition. Event *detectTimeout* requires MS module to detect and resolve short-term conflict within 10ms, otherwise timeout transition would be triggered.

State chart uses state and state transition to describe dynamic behavior model of system for event response, and is widely used for model programming. Comparing with state chart, automaton is formal to analyze recognizable language. As automaton is the foundation of state chart, it could accurately verify behaviors of class[20, 21].

### 3.3 Formal description based on PTA-OZ

As a graphical modeling language, MARTE works well in establishing the software model for RTES, but it has following deficiencies: 1) As defined in different ways, there are inconsistencies existing among abstract grammar, static semantics and dynamic semantics. 2) MARTE lacks reasoning authentication mechanism. These disadvantages limit MARTE’s application field and development, we therefore need to define precise and formal semantics for MARTE.

We proposed a formal integrated model called PTA-OZ[22], which uses Object-Z to describe the static semantics of MARTE and uses probability timed automata(PTA) to define its dynamic semantics. The formal integrated model could mathematically prove whether software models meet the requirements[23], and it could also increase the safety and correctness of software.

**Definition 1** A PTA-OZ is a tuple *PO* = (*A*, *I*, *L*, *P*, *OZ*), where:

- *A* is used to deal with inheritance, lists all parent class using *inherit* clause.
- *I* is the interface, and consists of channel declarations, which are provided and used by classes.
- *L* is local methods, which can only be accessed from the inside.
- *P* is a PTA that maps the state, event and transition of state chart to the attributes and operations of Object-Z.
- *OZ* is from Object-Z that includes a state schema, an initial schema and several operations.

We designed a transforming algorithm for the implementation of the formal model[24].By mapping the class and state chart of MARTE to Object-Z class and PTA expression in Object-Z format, we created the software model of flight control system through MARTE, and then translated into PTA-OZ model.

**Rule 1** In term of the basic transformation rules[25], we could define the transformation rule from MARTE model to PTA-OZ model. 1) MARTE class is translated to Object-Z class of PTA-OZ model. 2) The state chart of MARTE is mapped to PTA expression in Object-Z format. This rule is described as follows:

mapMARTEToPTA_OZ: MARTE→PTA_OZ

∀mar: MARTE•mapMARTEToPTA_OZ (mar) = {po: PTA_OZ |

∀mc:mar.class•∃oz:po.oz•oz = mapMARTEClassToOZ(mc)

∀ms:mar.statechart•∃pta:po.pta•pta = mapMARStateChartToPTA (ms)}

#### 3.3.1 Static class transformation

The class name of MARTE is mapped to the class name of Object-Z. The attributes of MARTE class are translated into state schemas. The association class is translated into the attribute of class. Interface operation invoked by class is translated into a channel schema of PTA-OZ. Interface operation offered by class is translated into a method schema.

**Fig 5:**
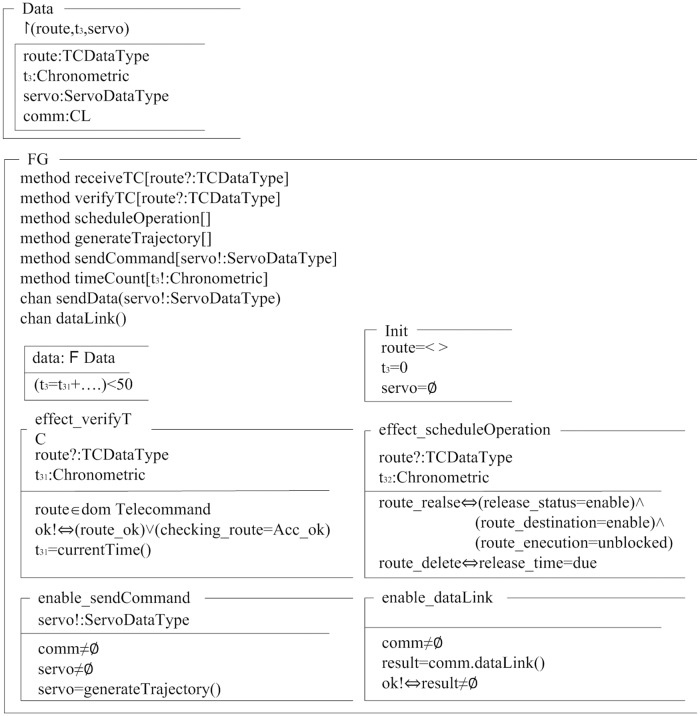
FG module described in Object-Z class. Object-Z is a formal language, it can be used to model the static model accurately.

Fig 5 showed a class of FG module, which was described in Object-Z format. Depending on class *FG*, association class *CL* was translated into attribute *comm* of class *FG*. The attributes *route*, *t*_3_, *servo*, *comm* of class *FG* were mapped to state schemas of PTA-OZ model. The operations like *verifyTc*, *scheduleOperation*, *sendCommand* provided by class *FG* were translated into methods of PTA-OZ model. The operations like *sendData*, *dataLink* invoked by the class *FG* were translated into channels of PTA-OZ model.

The processing time required by the invariance of the class *FG* should be no more than 50ms. We used *effect*+*operationname* to specify the operational effect, and used *enable*+*operationname* to express the operational trigger. For example, in *effect*_*scheduleOperation*, telecommand *route* releases if and only if the release status is *enabled*, the execution is unblock, and the destination can execute the command. The *guard* condition of operating *enable*_*sendCommand* is that connection *comm* has been established and servo command has been analyzed.

#### 3.3.2 Dynamic state chart transformation

As a class, the state chart can describe the behavior of class. In Object-Z, the behavior of class can create model in term of the attributes and operations of class. The attributes show the different statuses of the object, and the operations can change the value of the attributes. The state chart of MARTE consists of state, time event and transition. We thus mainly focus on these compositions to define transformation rules.

We used behavior attributes to create observable states model of object which value is boolean type. If true, object was in behavior state, and was regarded as an *active* state in MARTE. So each state should be mapped to a behavior attribute of Object-Z.

An event is the reception of a signal or the invoking request of an operation[26]. The response to a request can be modeled as receiving operation. Each event of state chart is mapped to the event acceptor operation of Object-Z. If an Object-Z operation was corresponding to a transition, we defined it as the triggered transition operation of event receives operation.

Time event is used to represent transition event triggered by time related factors and state transition in state chart triggered by time. The timing constraints of event in state chart are mapped to the clock constraints annotation on the edge of PTA. In Object-Z, we denoted state transition by comparing clock value with state invariance.

A transition represents either the change of object state or the execution of action. Due to that source state is the condition of transition and the target state is the result of transition, the value of source state is as the precondition of operation, and the value of target state is as the post condition of operation. The source state of transition is mapped to the initial state of behavior attributes in Object-Z, and the target states of transition are mapped to the termination states of behavior attributes.

A function *mapStateMachineToOZ*, which formally describes the transformation rules between state chart and Object-Z, is defined to translate state, time event and transition of state chart into the attributes and operations of Object-Z.

**Fig 6:**
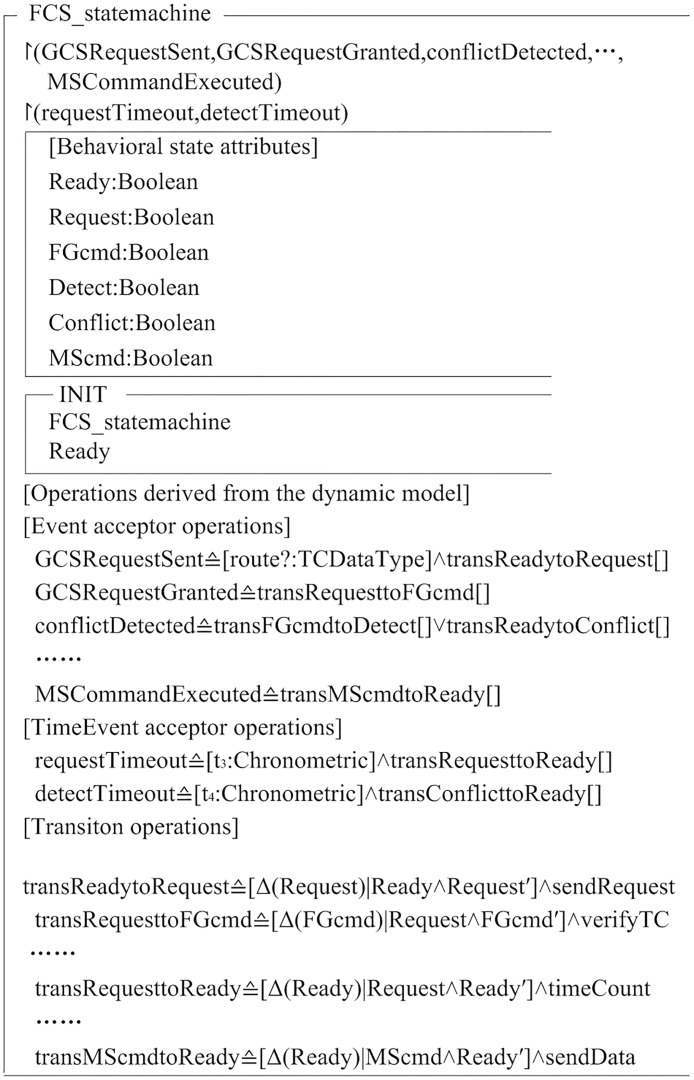
PTA expression in Object-Z format. Dynamic state transition can be described by the state schema in Object-Z.

Fig 6 showed a PTA expression in Object-Z format. The state is translated into the behavior attributes of Object-Z class. The initial state of state chart is expressed by the *Init* state schema in Object-Z class, and it is labeled *Ready*. An event is defined as different event receive operations. For example, event *GCSRequestSent* is defined as the operation of sending the *route* data, and causes the state to change from *Ready* state to *Request* state. Time event *requestTimeout* represents the transition event triggered by timeout, so that Its time can be realized by operation *timeCount*, and the state will be changed from *Request* state to *Ready* state. All the transitions belong to transition operations of Object-Z class. The behavior attributes of source state and target state are respectively defined as the precondition and post condition of transition operations. Transition operations contain all these effective activities. For example, the source state of transition *transRequesttoFGcmd* is *Request*, the target state is *FGcmd*, and effective activity is *verifyTC*.

## 4. Extracting Real-Time Specifications

The assurance of real-time requirement is the key of verifying software real-time, and is also the foundation for design, verification and realization of real-time reliability[27]. Most of the software real-time problems are caused by the inadequacy of acquiring real-time requirement. Flight control system is a hard real-time system, and it has strict deadlines on task. If the task could not satisfy the response time or response is not in time, it would lead to disastrous consequences.

The real-world software system developed according to natural language specifications is difficult to verify whether the resulting software satisfies the natural specifications[28]. For checking the software model, it is necessary to use a formal and precise symbol to represent the design specifications. Ogawa et al. proposed a goal-oriented analysis method of the requirement specification[29], which uses natural language to specify the specifications, then refines them into linear temporal logic (LTL) formulas and checks them through SPIN model checker. Therefore, model checking always uses LTL to specify properties to unambiguously describe the desired behavior of system. Each formula expresses a specific expectation of software behavior, a set of all formulas describe a consistent pattern of behavior.

### 4.1 Scenario-oriented requirement description

At the stages of design and verification, we need to identify the performance scenarios from end to end, which are usually extracted directly from requirements and used to evaluate the system response times[30]. The duration of a single processing or an end-to-end execution usually needs to be described. We can specify time information within the model by marking time-labeled UML2 interaction diagrams.

Scenario-oriented language is able to graphically describe the software requirements, and then be used to verify the properties of design model. The advantages are straightforward and visualization. Message sequence chart (MSC) is widely used in the development field of industry software by International Telecommunication Union(ITU) as the standard and the description language of communication behavior of real-time system. However, MSC is only used to describe the causal relationships among messages, but explain the time partial order constraints of behavior, and clearly distinguish specifications from executable requirements[31, 32]. These drawbacks of MSC limit its expression ability and application. Based on the extension of MSC, Werner Damm and David Harel proposed live sequence chart (LSC) [31], which distinguishes the existential scenario and the universal scenario of system. If the condition of universal scenario was true, the system must execute the scenario described in sequence chart, meaning the universal scenario is suitable to specify the activity of scenario.

Yves Bontemps et al. applied an extension of LSC to air traffic control system[33],and specified scenario-oriented requirements by associating an instance with a class. But LSC still adopts timing constraints in MSC, such as timer and delay interval, turning out that this application is limited.

MARTE extends sequence diagram, and defines timing constraints on sending and receiving events, and could record the start and end time, and also uses value specification language (VSL) to specify timing constraints among events. For overcoming the shortcomings of LSC in time property, we adopted MARTE to enrich the expressive power of LSC, and proposed TLSC method, which could intuitively describe time-enriched property and timing partial order relation of behaviors.

For overcoming the drawbacks of LSC in time property, we proposed TLSC method to intuitively describe time-enriched property and timing partial order relation of behaviors by using value specification language(VSL) to specify timing constraints among events and to extending sequence diagram by using MARTE. The extension by MARTE is that adding timing constraints to sending and receiving events for recording the start and end time.

We still adopted the property *on* of «*TimedProcessing*» stereotype to associate clocks with current TLSC. Sequence diagram is used to specify the interaction behavior among objects, which may be restricted by time factors in time trigger architecture, thus we need to introduce time observation to describe timing constraint. Time observation offers a method to acquire execution time and duration of system. «*TimedInstantObservation*» stereotype acquires the start or end instant and uses operation @*t* to store acquired time in variable *t*. «*TimedDurationObservation*» stereotype can be used to express the duration of event, using {*t*, *t*+*d*} to denote that an event starts at *t* time and ends at *t*+*d* time.

**Fig 7:**
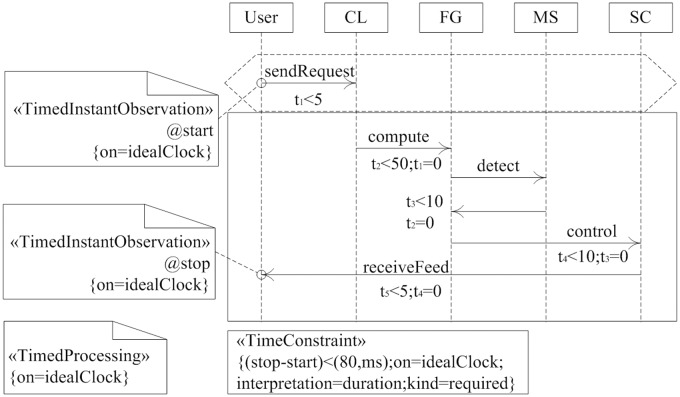
Scenario-oriented requirement for flight control software. MARTE time model is used to describe the requirement. With timing constraints.

Fig 7 showed a scenario-oriented end-to-end requirement for flight control software. In order to reference ideal clock, we use «*TimedInstantObservation*» stereotype of MARTE to express time observation. Similarly, we defined a time observation of UML to associate with receive events of control message, and referred to the same ideal clock. The rules of execution time among modules of flight control software are that the delay time for receiving telecommand is less than 5 *ms*, the calculating time of flight path is less than 50 *ms*, the response time of conflict detection is less than 10 *ms*, the execution time of servo control is less than 10 *ms*, the output delay time of telemetry is less than 5 *ms*. That is to say, the time from sending telecommand to receiving telemetry is no more than 80 *ms*. Thus, we need to define a timing constraint, which shows that the duration between time observation events *stop* and *start* is less than 80 *ms*.

### 4.2 Monitoring real-time specification

In the process of software design, it is difficult to design a bug-free system, so we need to employ model checking to find the bugs, which can be used to verify whether software model meets design specification. It is difficult to write bug-free specifications, since we do not know whether the system specification could fully capture the design expectation of software.

Although graphical TLSC could intuitively express timing partial order relation of behaviors, and is suited to describe customer requirement by software engineer, it could not be used to verify the system specification. In the scenario-oriented software engineering, temporal logic is widely applied to describe the software requirements, but it is not realistic to require software engineer to intuitively use temporal logic formula to specify software requirement. Therefore, we tried to extract time property formula from software requirement of flight control system described by TLSC. Firstly, we gave the formal definition of TLSC[34].

**Definition 2** A TLSC is a tuple TLSC = <*I*, *Loc*, *E*, *C*, *δ*, *Mode*, *inv*, *μ*>, where

- *I* is a set of instances.
- *Loc* is a set of locations.
- *E* is a set of events, and it contains two events condition *Con* and message *Msg*, that is, *E* = *Con*U*Msg*.
- *C* is a finite set of clock variables.
- *δ*: *E*→*Loc* is an event mapping function which maps each event to a location.
- *Mode*: *E*→{*cold*, *hot*} is a behavior mapping function which identifies each event with a provisional or mandatory behavior.
- *inv*:*Loc*→*Φ*(*C*) is a timing constraint mapping function, by which each location ‹*i*,*l*› is given assigns timing constraint *inv*(*l*) called location invariance.
- *μ*:*E*→*Φ*(*C*) is a time interval mapping function, it defines the time interval of event occurrence.

Trace-based semantic adopts finite or infinite state sequence to describe the relation of state transition. Using the form of trace-based semantic, precise meaning of software behavior could be reflected, and temporal logic formula could be extracted from TLSC. Here we gave the trace-based semantic of universal chart in TLSC.

**Definition 3** Let a *CUT* sequence *r =* (*cut*_0_,*v*_0_), (*cut*_1_,*v*_1_),…, (*cut*_*k*_,*v*_*k*_) be an execution of TLSC, where *cut*_*i*_ denotes a mapping of current locations of all the instances, and *v*_*i*_ denotes the clock interpretation of current state. *cut*_0_ is the starting point and clock interpretation *v*_0_ = 0, *cut*_*k*_ is the terminal point, and (*cut*_*i*_,*v*_*i*_) = *succ*((*cut*_*i*-1_,*v*_*i*-1_),<*i*, *l*_*i*_>) (*i =* 0,1,…,*k*-1). The set of all runs is denoting to *Run*s. We use *r*^*k*^ = {*r*|∀*r*∈*Runs*^|*r*| = *k*} to denote the sub path of run *r* with *k*-path, and use *Path*(*cut*_*i*_,*v*_*i*_) = {*r*|∀*r*∈*Runs*^*cut*_0_ = *cut*_*i*_} to denote a path of a run starting at (*c*_*i*_,*v*_*i*_). An execution trace, which is the trace of a *CUT* sequence, is denoted by *π = trace*(*r*)

$$cut_{0}\overset{\pi_{0}}{\to }cut_{1}\overset{\pi_{1}}{\to }\cdots \overset{\pi_{k-1}}{\to }cut_{k}$$

Then *π* = *π*_0_, *π*_1_,…, *π*_*k*-1_ denotes the events that trigger the state transition, and

$$\pi_{i}=\{\begin{array}{ll} \delta (i,l_{i}) & if(cut_{i},v_{i})=succ((cut_{i-1},v_{i-1}),\langle i,l_{i}\rangle )\\ \varepsilon & else \end{array}$$

In term of execution trace, we define the trace-based language of TLSC *tl*, that is

$$L=\{\pi |\exists r\in Runs(tl)\wedge r=(cut_{0},v_{0}),\ldots ,(cut_{k},v_{k})\;s.t.\;\pi =trace(r)\}$$

In the universal chart, all runs must satisfy the given scenario. If executions *r* of TLSC are in the same sub chart, the relation between formulas is logical conjunction. If executions *r* are in the different sub charts, the relation is logical implication. Algorithm 1 showed that temporal formulas corresponding to different message in sub chart were combined into an algorithm about ACTL formula.

**Algorithm1** combinedGenerating(*ϕ*_*msg*1_,*ϕ*_*msg*2_)

1: **if** type(*ϕ*_*msg*1_) = pre-chart **then**

2:  **if** type(*ϕ*_*msg*2_) = pre-chart **then**

3:   *ϕ* = ¬*ϕ*_*msg*2_U (*ϕ*_*msg*1_(X*ϕ*_*msg*2_))

4:  **elseif** type(*ϕ*_*msg*2_) = main-chart **then**

5:   *ϕ* = *ϕ*_*msg*1_→(X*ϕ*_*msg*2_)

6:  **endif**

7: **elseif** type(*ϕ*_*msg*1_) = main-chart **then**

8:  **if** type(*ϕ*_*msg*2_) = pre-chart **then**

9:   *ϕ* = *ϕ*_*msg*1_

10:  **elseif** type(*ϕ*_*msg*2_) = main-chart **then**

11:   *ϕ* = ¬*ϕ*_*msg*2_U (*ϕ*_*msg*1_(X*ϕ*_*msg*2_))

12:  **endif**

13: **endif**

14: return(*ϕ*)

Universal chart can be used to express the mandatory scenario. If the event in pre-chart was activated, there must have response event in main chart. Hence in term of Algorithm 1, we could get the ACTL formula corresponding to TLSC as follow

$$\begin{array}{l} AG((\underset{\forall p_{i}\in Msg_{p}}{\wedge }{\phi^{\prime }}_{p_{i},succ(p_{i})}\wedge \underset{\begin{array}{l} \forall m_{i}\in Msg_{m},\\ p_{j}=Max_{p}(i_{j}) \end{array}}{\wedge }{\phi^{\prime }}_{p_{j},m_{i}}\wedge \underset{\begin{array}{l} \forall p_{i}\in Msg_{p},\\ p_{j}=Max_{p}(i_{j}) \end{array}}{\wedge }\neg \chi_{p_{i},p_{j}})\\ \;\to (\underset{\forall m_{i}\in Msg_{m}}{\wedge }{\phi^{\prime }}_{m_{i},succ(m_{i})}\wedge \underset{m_{j}=Max_{m}(i_{j})}{\wedge }AF^{inv+u}m_{j}\wedge \underset{\begin{array}{l} \forall e_{i}\in Msg,\\ m_{j}=Max_{m}(i_{j}) \end{array}}{\wedge }\neg \chi_{e_{i},m_{j}}))\; \end{array}$$

Where *Msg* is the set of messages, *succ*(*m*) denotes the immediate successor of message *m*. ${\phi^{\prime }}_{e_{i},e_{j}}=\neg e_{j}\cup e_{i}$ is order attribute and it shows that message *e*_*j*_ will not occur until *e*_*i*_ occurs. $\chi_{e_{i},e_{j}}=(\neg e_{i}\wedge e_{j})\cup (e_{i}\wedge X((\neg e_{i}\wedge e_{j})\cup e_{i}))$ is uniqueness property and denotes that message *e*_*i*_ occurs twice before *e*_*j*_. We can also use optimization strategies to improve the algorithm performance[35].

Using Algorithm 1, we could extract temporal logic formula of real-time property from TLSC for flight control system as showed in Fig 7. After sending message *sendRequest* to UAV from GCS, each module began to work, and FG module computed the flight path, MS module detected conflict, and SC module sent servo command. We could get the temporal logic formula of real-time property from scenario-oriented TLSC as follow

$$AG(A(\neg compute\wedge \neg detect\wedge \neg control)\cup sendRequest)\to AF^{t<80}receiveFeed)$$

## 5. Real-Time Reliability Analysis

In the verification of PTA-OZ model, we could check the correctness of grammar and type in Object-Z part, and make sure that all the operations strictly use state model. By using the existing formal tools Z/EVES and PVS, specification could be checked and analyzed in Z form. We could automatically extract corresponding burden of proof for Object-Z specification according to a certain rule, and then test by inputting them to Z/EVES. Through strictly type examination, we could analyze specifications in Object-Z form, and locate the inconsistent information between specifications and requirements.

However, Object-Z is only suitable for data refinement, and does not support the structure similar to program language. Hence, there is semantic gap between Object-Z and program language, while PTA-OZ model can overcome the shortcoming and realize operation refinement through verifying the correctness of operation.

PTA model could be used to express the state transition diagram of flight control system. We could create mathematics model of real-time reliability by applying Markov process to achieve the state transition probability matrix of system. There are three status types of real-time reliability: *up* denotes that system is in working, *down* denotes that system shuts down, *danger* denotes that some transient failures have occurred but have not yet caused system shutdown.

There are various kinds of sensors in UAV for receiving signal, data or command. The performance of sensors is a gradual recession process, thus reduce the reliability of hardware devices. The failure of sensors will cause the software failure of FG module. Though the reliability assessment of hardware devices, the reliability of sensors will reduce after working 1000 *h*, and will down to 0 after working 1500 *h*. In flight control system, software module will reboot to rectify the transient fault. If the system was in sensor failure, FG module was unable to read data from the sensor, meanwhile the system would be forced to skip the current cycle. If the number of skipped cycles exceeded a threshold value, then flight control system would fail and emergency instructions or self-destruct would start.

**Fig 8:**
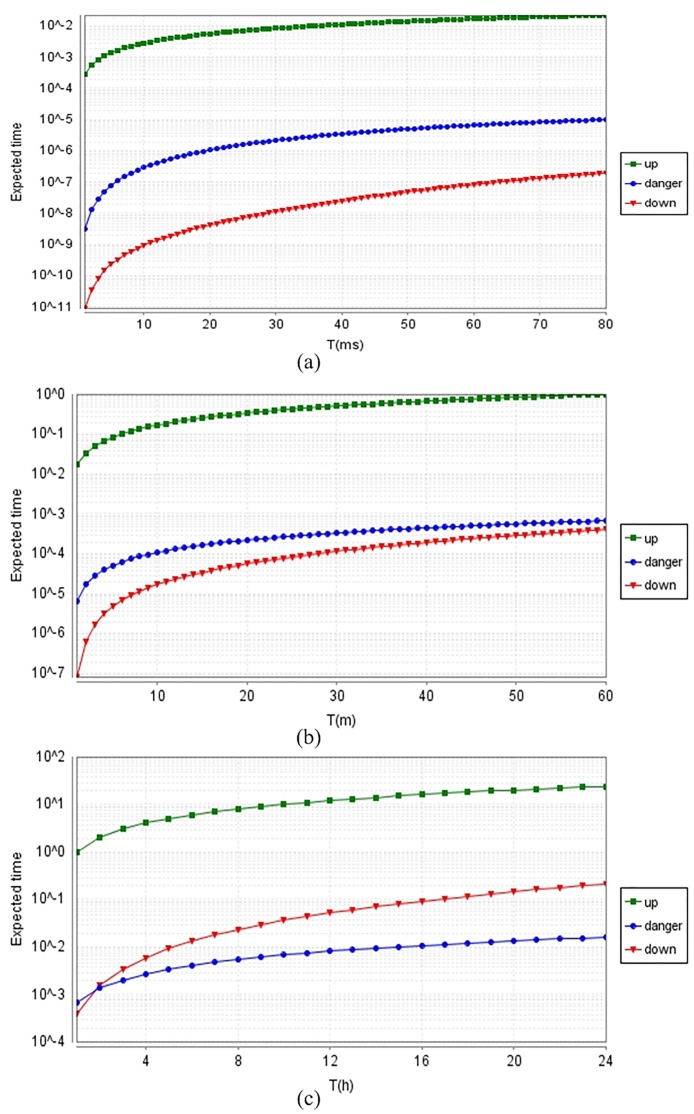
The expected time of each status within T unit time described in logarithm form. (a) The expected time within 80ms. (b) The expected time within 60m, (c) The expected time within 24h. (a)Within 80ms, the probability of system shutdown is smaller. (b) As time goes on, the danger status goes up. The UAV is fit to carry out a short-term mission. (c)The reliability of hardware determines UAV whether can perform tasks for a long time.

Fig 8 showed the expected time of each status within T unit time described in logarithm form. Since the requirement of total time from sending telecommand to receiving telemetry is no more than 80 *ms*, Fig 8(a) showed the expected time of different system states within 80 *ms*. Fig 8(b) showed the expected time of different system states within 1*h* when UAV carries out a short-term mission. The expected time in failure status gradually increases, but it is still less than the working time. Fig 8(c) showed that the expected time of *down* status will increase significantly while UAV carries out a long-term mission. As shown in Fig 8, the failure status of software caused by hardware fault would gradually increase along with the execution time increase. Thus we could improve the design method of software through enhancing the reliability of hardware.

The integrated framework for UAV flight control development can execute real-time simulation[1]. It adopts real-time operating system, does real-time monitoring by GCS, and executes the embedded software code in real-time. Compared with similar method[1], we can accurately calculate the expected time of each status. The result showed that UAV is highly reliable when it carries out a short-term mission and it is not suiteable for a long-term mission.

**Fig 9:**
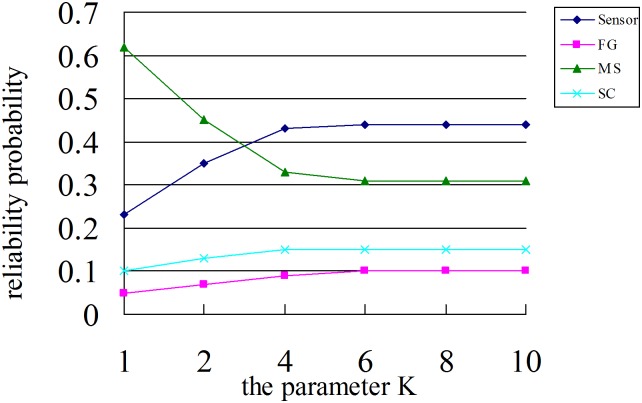
The reliability probability of each processor. With the increasing K value, the reliability probability became stable.

The system reliability depends on the threshold value K, Table 1 showed the expected time of system states with danger and up. When the value of K increases, the expected time both increase. The increasing of expected time in up is significantly higher than in danger. The different value of K also effects the system reliability. The reliability probability of processor-in-the-loop was given in Fig 9. The increasing value of K makes the reliability probability to stabilize.

**Table 1:** The Expected time in states “danger” and “up”.

K	Expected time	
	danger(hrs)	up(days)
1	0.29	420.78
2	0.39	559.40
4	0.45	651.25
8	0.46	664.53
16	0.46	664.74

## 6. Conclusions

Compared with existing integrated framework[1, 36, 37], we used graphics modeling language MARTE to create software model of flight control, and transformed static structure and dynamic behavior models into formal integrated framework PTA-OZ through defined transformation strategies. We specially focused on the model and verification of time property, which is not considered by most integration framework. The different processes such as analysis, modeling, transforming and verification in proposed framework are tightly-coupled.

For modeling and verification of the real-time reliability of UAV flight control system, we have mainly done following works. Combined with DO-178C standard, we studied model-based method, object-oriented technology and formal method in the framework of software development, and propose a formal PTA-OZ model, which can formalize MARTE model in an object-oriented way by transformation rules. To eliminate the difference in the description of the real-time property formula among software engineers, we proposed an extracting method of temporal logic formula from TLSC, which could automatically generate real-time specification. By verifying the real-time reliability of software model, we could analyze its status type at different time periods.

In order to meet the airworthiness certification, we proposed a design, development and validation framework for UAV flight control system. To begin with, we used MARTE to create the time property model, then translated it into PTA-OZ model. Eventually we could analyze and verify the real-time reliability of UAV flight control system by existing formal verification tools.

## Supporting Information

S1 Fig The reliability probability of each processor. With the increasing K value, the reliability probability became stable.
(TIF)

S1 Table The reliability probability for different value of K. (DOC)
