# Supplementary figures and images for: Real-Time Reliability Verification for UAV Flight Control System Supporting Airworthiness Certification
Source: PLoS One. 2016 Dec 5;11(12):e0167168. doi: 10.1371/journal.pone.0167168 (PMC5137893; doi:10.1371/journal.pone.0167168)

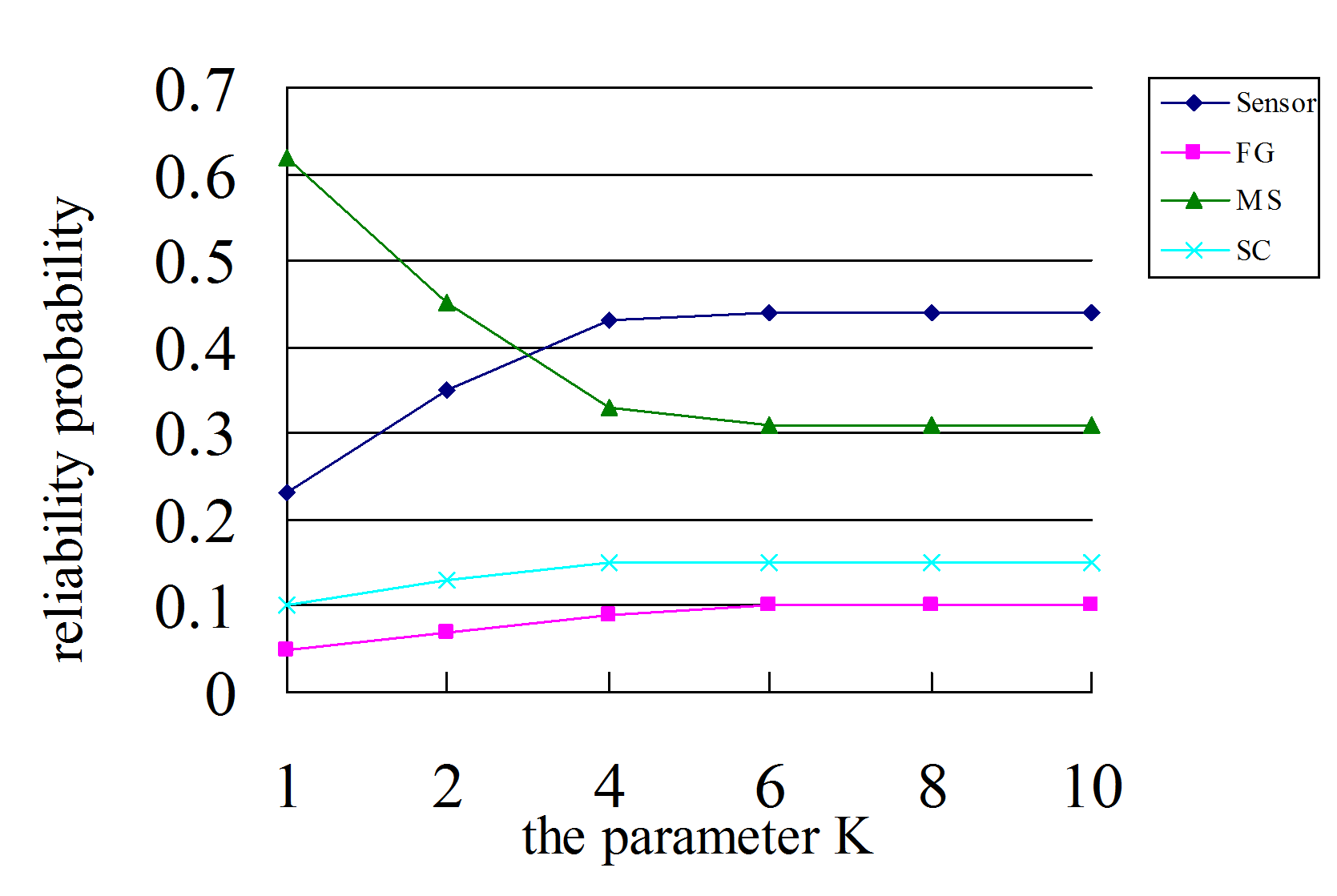

Supplement: S1 Fig — With the increasing K value, the reliability probability became stable. (TIF) [file pone.0167168.s001.tif]
