# Supplementary material for: Real-Time Reliability Verification for UAV Flight Control System Supporting Airworthiness Certification
Source: PLoS One. 2016 Dec 5;11(12):e0167168. doi: 10.1371/journal.pone.0167168 (PMC5137893; doi:10.1371/journal.pone.0167168)
Supplement: S1 Table — (DOC) [file pone.0167168.s002.doc]

**S1 Table. The reliability probability for different value of K**

| K | 1 | 2 | 4 | 6 | 8 | 10 |
| --- | --- | --- | --- | --- | --- | --- |
| Sensor | 0.23 | 0.35 | 0.43 | 0.44 | 0.44 | 0.44 |
| FG | 0.05 | 0.07 | 0.09 | 0.10 | 0.10 | 0.10 |
| MS | 0.62 | 0.45 | 0.33 | 0.31 | 0.31 | 0.31 |
| SC | 0.10 | 0.13 | 0.15 | 0.15 | 0.15 | 0.15 |
